# Adaptive Machine Learning Based Distributed Denial-of-Services Attacks Detection and Mitigation System for SDN-Enabled IoT †

**DOI:** 10.3390/s22072697

**Published:** 2022-03-31

**Authors:** Muhammad Aslam, Dengpan Ye, Aqil Tariq, Muhammad Asad, Muhammad Hanif, David Ndzi, Samia Allaoua Chelloug, Mohamed Abd Elaziz, Mohammed A. A. Al-Qaness, Syeda Fizzah Jilani

**Affiliations:** 1 School of Computing, Engineering and Physical Sciences, University of the West of Scotland, Glasgow G72 0LH, UK; muhammad.aslam@uws.ac.uk (M.A.); david.ndzi@uws.ac.uk (D.N.); 2 School of Cyber Sceince and Engineering, Wuhan University, Wuhan 430079, China; yedp@whu.edu.cn; 3 State Key Laboratory of Information Engineering in Surveying, Mapping and Remote Sensing (LIESMARS), Wuhan University, Wuhan 430079, China; aqiltariq@whu.edu.cn (A.T.); alqaness@whu.edu.cn (M.A.A.A.-Q.); 4 Department of Computer Science, Nagoya Institute of Technology, Nagoya 466-8555, Japan; a.muhammad.799@nitech.ac.jp; 5 Department of Computer Science, COMSATS University of Islamabad, Wah Cantt 45550, Pakistan; hanif-cui@ciitwah.edu.pk; 6 Department of Information Technology, College of Computer and Information Sciences, Princess Nourah bint Abdulrahman University, P.O. Box 84428, Riyadh 11671, Saudi Arabia; 7 Department of Mathematics, Faculty of Science, Zagazig University, Zagazig 44519, Egypt; abd_el_aziz_m@yahoo.com; 8 Department of Physics, Aberystwyth University, Aberystwyth SY23 3FL, UK; sfj7@aber.ac.uk

**Keywords:** Internet of Things, Distributed Denial-of-Services, network security, software defined networking, adaptive machine learning, detection, mitigation

## Abstract

The development of smart network infrastructure of the Internet of Things (IoT) faces the immense threat of sophisticated Distributed Denial-of-Services (DDoS) security attacks. The existing network security solutions of enterprise networks are significantly expensive and unscalable for IoT. The integration of recently developed Software Defined Networking (SDN) reduces a significant amount of computational overhead for IoT network devices and enables additional security measurements. At the prelude stage of SDN-enabled IoT network infrastructure, the sampling based security approach currently results in low accuracy and low DDoS attack detection. In this paper, we propose an Adaptive Machine Learning based SDN-enabled Distributed Denial-of-Services attacks Detection and Mitigation (AMLSDM) framework. The proposed AMLSDM framework develops an SDN-enabled security mechanism for IoT devices with the support of an adaptive machine learning classification model to achieve the successful detection and mitigation of DDoS attacks. The proposed framework utilizes machine learning algorithms in an adaptive multilayered feed-forwarding scheme to successfully detect the DDoS attacks by examining the static features of the inspected network traffic. In the proposed adaptive multilayered feed-forwarding framework, the first layer utilizes Support Vector Machine (SVM), Naive Bayes (NB), Random Forest (RF), k-Nearest Neighbor (kNN), and Logistic Regression (LR) classifiers to build a model for detecting DDoS attacks from the training and testing environment-specific datasets. The output of the first layer passes to an Ensemble Voting (EV) algorithm, which accumulates the performance of the first layer classifiers. In the third layer, the adaptive frameworks measures the real-time live network traffic to detect the DDoS attacks in the network traffic. The proposed framework utilizes a remote SDN controller to mitigate the detected DDoS attacks over Open Flow (OF) switches and reconfigures the network resources for legitimate network hosts. The experimental results show the better performance of the proposed framework as compared to existing state-of-the art solutions in terms of higher accuracy of DDoS detection and low false alarm rate.

## 1. Introduction

The recent advancement of Internet of Things (IoT) represents a paradigm shift of modern global communication infrastructure [1]. It revolutionizes the many aspects of the urban living of smart cities by enabling the intercommunicability of the smart communication systems. As more smart computing devices such as smartphones, wristbands, sensors, and actuators equipped with significant computational powers and data processing operated by human users integrate into IoT infrastructure, more useful applications of IoT emerge [2,3]. Many internet-oriented smart city applications of IoT also maximize the production of IoT devices due the seamless integration of the 5G network. However, recent security reports indicate that hackers are successfully penetrating through loosely guarded IoT devices [2,4]. The IoT network contains thousands of relatively less secured devices, which formulates the feasible environment to launch DoS, DDoS, brute force, and TCP SYN/UDP flooding against network devices. Particularly, the implementation of botnets is increasing the volume of DDoS attacks and millions of IoT devices are facing such attacks [5,6].

**Figure 1:**
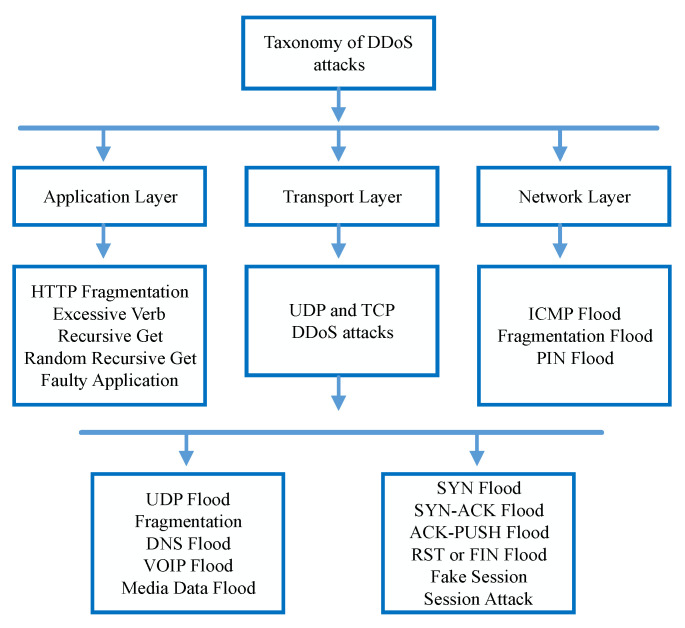
Taxonomy of TCP/IP model DDoS attacks.

Flooding-based DDoS attacks cause consistent threat towards the smart industrial service providers of IoT. Frequently, such attacks follow the networking protocols and cause extensive network traffic and drain the network resources. Such intense penetrations of DDoS attacks result in the expensive exhaustion of servers and individual hosts’ communication capacity to respond to legitimate users. Usually, DDoS attackers explore UDP Flood, PING Flood, SYN Flood, and HTTP Flood to launch DDoS attacks [7,8,9]. According to the TCP/IP model, the taxonomy of well-known DDoS attacks is shown in Figure 1. Moreover, many spoofing tools and sophisticated online services are available to launch and hide the identity of the DDoS attackers, so network security systems become more fragile to such frequently launched attacks [10,11]. Until now, most of the major content providers such as Youtube, Facebook, Twitter, and Amazon have experienced service unavailability due to DDoS attacks. Similarly, IoT network operators also lack the sophisticated security tools to develop completely immune communication network from DDoS attacks [12,13].

Attackers on network security have advanced to a high level of technical competence. For normally built dispersed networks, they can modify packet header data and execute legitimate extensive service requests on specific workstations or servers [14,15]. However, the central coordination system of SDN promises the extensive traffic analysis at the control plane for forwarding plane network traffic to achieve the appropriate security response towards different network security threats [12,16,17,18]. Such early success of SDN integration has diverted the researcher’s attention towards the possible advancement of network security to enable the successful detection and mitigation of DDoS security attacks [12,18,19,20]. Some key features of SDN-enabled networks, such as separation of the control plane and data plane, centralized network topology view, dynamic system reconfigurations, and the programmability of the network devices, play a vital role in enabling practical security mechanisms. These features of SDN integration play an essential role in the development of intelligent SDN-enabled intrusion detection and prevention systems [21,22,23,24].

Recently, machine learning classifier algorithms are being integrated to upgrade the Intrusion Detection Systems and Intrusion Prevention Systems. Machine learning classification and regression algorithms enhance the intelligence of the system to differentiate the anonymous traffic from normal network traffic [25,26,27]. Many phishing and DDoS attacks are being classified through adaptive machine learning frameworks. Despite these efforts, those frameworks need a central reconfiguration system to develop an effective mitigation system for online network traffic of IoT [1,28]. The well-known algorithms of those frameworks include Support Vector Machine (SVN), Gaussian Naive Bayes (GNB), Logistic Regression (LR), k-Nearest Neighbors (KNN), and Random Forest (RF) [29].

To this end, we propose the AMLSDM framework to implement adaptive machine learning classification to detect and mitigate the DDoS attacks for SDN-enabled IoT networks. The proposed AMLSDM framework contains the following building blocks: training of multilayered feed-forwarding approach based adaptive machine learning model over the environment-specific dataset, feature extraction of real-time SDN-enabled IoT network, inspection and detection of DDoS attacks, and DDoS mitigation system. Our proposed model is the extension of our conference paper [30]. In the proposed AMLSDM framework, the feature extraction and inspection modules execute over the OF switches, which further update traffic entries towards the SDN controller. In particular, the DDoS detection is accomplished over OF switches, and DDoS mitigation is archived over the SDN controller response of congestion control. Furthermore, the SDN controller reconfigures the communication paths based on the reports of OF switches. In this way, the proposed AMLSDM framework achieves the detection and mitigation of DDoS attacks. To be precise, we develop the adaptive multilayered feed-forwarding approach of the adaptive machine learning classification model by the combination of the SVM, GNB, RF, KNN, and LR classifiers with support of the Ensemble Voting (EV) model. The major contributions of the proposed framework are as follows:

1. We develop a novel AMLSDM framework that supports feed-forwarding through adaptive machine learning classification for DDoS detection and mitigation system for SDN-enabled IoT. We design the module of the adaptive machine learning model with the support of the EV classification model, which accumulates the measurements of SVM, GNB, RF, KNN, and LR machine learning classifiers.
2. We develop the inspection module for feature extraction of real-time network traffic and compute the entry for current network flow. Then, we integrate the trained module of the adaptive machine learning model with an environment-specific dataset for real-time network traffic flow to detect the DDoS attacks.
3. We configure two custom topologies of SDN enabled IoT networks for experimentation in a virtual environment using Mininet, OF switches using Open vSwitch, remote POX, and Floodlight SDN controllers. The sFlow network flow analyzer is also used to record real-time statistics. On the LINUX machines that have been switched to OF switches, the Host sFlow Daemon is installed. The Sflow-RT run on the monitoring server that collects the real-time statistics of the OF switches.
4. We launch the different DDoS attacks to test the detection and mitigation capability of AMLSDM framework and achieves promising simulation results.

The rest of the paper is organized as follows: Section 2 provides a brief review of related work to identify the core problem of existing solutions. Section 3 presents the technical details of the primary building blocks of our proposed AMLS-DM framework. In Section 4, we propose the AMLSDM framework and provide the detailed work-flow of the DDoS detection and mitigation process. In Section 5, we present the simulation results obtained through extensive experiments. In Section 6, we conclude the proposed research contribution and defines the future research direction.

## 2. Related Work

SDN provides networking applications for all the wired and wireless networks, including IoT. Recently, many research papers have been published, and many industrial solutions are also available, in which SDN is being implemented for wireless networks, including IoT networks, to enhance the security features of the IoT networks. The extensive deployment of IoT-equipped devices has reached billions after the emergence of intelligent civilian healthcare and military surveillance applications. Due to limited computational and communication resources, these IoT-equipped networks have experienced millions of attacks due to a lack of security concerns in the design of the devices [3,8]. However, SDN integration within IoT networks reduces the computational burden and adds security enhancements due to the central reconfigurations system of the control layer [22,23,24]. Machine learning algorithms based on DDoS detection systems further strengthen the network security of SDN-enabled IoT networks. Many advanced solutions provide successful intrusion detection with the support of machine learning. Such solutions are widely deployed for different applications such as auditing applications and smart grid and IoT [31,32,33] Some of the solutions provide virtual machine learning-based DDoS detection systems for SDN-enabled networking, but still demand significant enhancements to develop industrial level security solution [28].

Recent literature has proposed certain network-based strategies based on SDN solutions, with the lead foundational framework to go forward in fixing the issue of DDoS attacks. In [34], a machine learning-based DDoS detection system for SDN-enabled IoT is proposed, called LEDEM. The detection of LEDEM is based on a semi-supervised machine learning algorithm. The major problem with this proposed model is the lack of adaptiveness, as LEDEM utilizes only a single classification algorithm and is unable to deal with DDoS attacks of diverse nature. In [35], a general framework for software-defined Internet of Things (SD-IoT) is proposed. SD-IoT analyzes the network traffic of IoT and, based on network characteristics, provides DDoS detection. SD-IoT also faces the limitation of limited machine learning classification capability. In [18], the traffic flow features are used to detect DDoS attacks, which results in a lightweight solution. The flow collector, feature extractor, and classifier modules are the three components of this suggested model. This solution demonstrates effective DDoS information extraction with relatively low overhead in comparison to other methods. However, for busy network traffic, this method is insufficient, necessitating the use of a more complex security system. In [19], the identification of DDoS attacks is carried out using content-based prospective attackers; this technique is known as Content-Oriented Networking Architecture (CONA).

In CONA, the requests pattern is monitored, and servers use content-based thresholds to reduce DDoS effects. Some review work [36,37] indicates the new trends and challenges of SDN-enabled IoT DDoS detection and mitigation solutions.

To this end, in this paper, we propose the AMLSDM framework, which is an adaptive machine learning-based DDoS detection and mitigation system, to deal with the limitations of existing solutions. Our solutions utilize the wireless applications of SDN with the help of available setting for wireless networking in Mininet. Most importantly, at forwarding layers, we have associated IoT devices in our simulation’s configurations. Furthermore, we have selected the most popular and successful machine algorithms, i.e., SVM, GNB, RF, KNN, and LR, for DDoS detection during the implementation of the EV model. These machine algorithms are widely accepted and cited by the recent research work for DDoS detection in IoT networks.

## 3. System Models

In this section, we first present the network topology and then discuss machine learning classifiers. Lastly, we briefly define the threat model, which is a major motivation to the proposed framework.

### 3.1. SDN-Enabled IoT Network Topology

**Figure 2:**
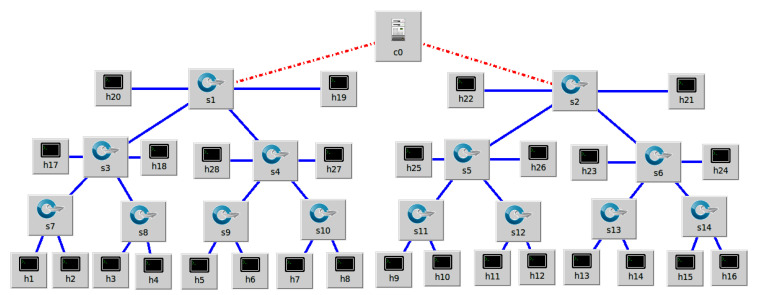
Custom SDN-enabled IoT network topology with POX Controller.

**Figure 3:**
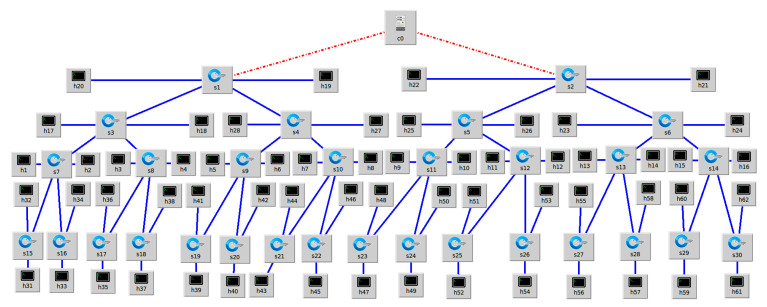
Custom SDN-enabled IoT network topology with floodlight Controller.

The SDN-enabled IoT network topology is launched by Mininet emulator, which is being coordinated to remote SDN controllers of POX and floodlight at the control layer. The deployed IoT hosts are connected with OF switches at the forwarding plane. The primary responsibility of these OF switches is to notify the SDN controller of any new incoming communication requests. In practice, OF switches send DDoS attack reports to the SDN controller, as well as the congestion rate over available communication lines. The DDoS built-in congestion control system is reactively performed by the SDN controller. Initially, OF switches execute the trained adaptive machine learning classification designed by combining SVM, GNB, RF, KNN, and LR at the EV model. For genuine host requests in mitigation response, the SDN controller additionally controls alternate path computation based on link flow congestion statistics and operational ports. OF switches function as intermediary devices, performing path returns and reporting statistics in bytes on a regular basis in accordance with the flow rules. Meanwhile, the SDN controller keeps track of the network view and the hierarchical structure of received statistics in a hash table. The computed alternative paths are also classified by the SDN controller using the network path congestion index for each path. The estimated network path congestion index of alternative paths is inversely proportional to the number of alternative paths installed by the SDN controller over OF switches. Figure 2 and Figure 3 present the two SDN-enabled IoT network topologies, which we also use for experiment purpose in this paper. In network topology, we design a custom SDN-enabled IoT network topology with a POX Controller. Meanwhile, in the second network topology, we design an SDN-enabled IoT network topology with a Floodlight Controller. In both topologies, we have OpenFlow switches at the forwarding plane which are further connected with IoT devices. Meanwhile, at the control plane, we have SDN controllers to mitigate the DDoS attacks.

### 3.2. Machine Learning Classifiers

Here, we briefly discuss the SVM, NB, RF, KNN, LR, and EV classification algorithms.

#### 3.2.1. Support Vector Machine (SVM)

The SVM algorithm belongs to supervised hyperplane-based classifiers algorithms, which are discriminative and parametric classifiers [38]. Support Vector Machines (SVM) are built for binary classification in all hyperplane-based situations and do not support multi-class classification jobs natively. Finding a hyperplane with the greatest margin and algorithm is recommended in order to have the most margin with the smallest number of points. The major goal is to maximize the minimum distance $w^{*}$.

(1) $$w^{*}=arg_{w}max\left[min_{n}d_{H}\left(\phi \left(x_{n}\right)\right)\right]$$

where $d_{H}(\phi \left(x_{n}\right)$ is distance of a hyperplane equation which is basically derived from distance of any line ax+by+c=0 from a given point, say, (x0,y0) is given by *d*. So, now that we know what we’re trying to do, we can use the point from the positive group in the hyperplane equation to get a value greater than 0 when making predictions on the training data, which was binary classified into positive and negative groups, Mathematically:

(2) $$w^{T}\left(\phi \left(x\right)\right)+b>0$$

Predictions from the negative group in the hyperplane equation would give negative value as:

(3) $$w^{T}\left(\phi \left(x\right)\right)+b<0$$

These hyperplane-based classifiers provide classical classification by maximizing the margin iteratively perceptron learning, Fisher linear discriminant analysis, and least-squares optimization. We utilize the hyperplane-based classifiers of SVM and Logistic Regression for classifying the SDN-enabled IoT network traffic to identify the DDoS attacks. Mainly, the SVM classifier learning model trains the model by using multiple kernel functions of polynomial functions, radial basis functions. In the classical setting of SVM, from a set of training data points, the SVM algorithm represents them in a space; it maps them by categories and divides them by the separating hyperplanes. The decision boundary is maximized; when new data points arrive based on the nature of the point, it categorizes the data into the clusters previously formed. Thus, the SVM algorithm can successfully differentiate the exact nature of the flow of traffic in both normal and DDoS scenarios [39].

#### 3.2.2. Naive Bayes (NB)

NB works on the principle of independent variable comparison and finds the relationship between these independent variables [40]. This ML algorithm works on a theorem of Bayes in which attributes are true. It is simple to construct as there is no parameter evaluation on the algorithm. This allows it to work on very large datasets. The Bayes theorem defines the following relationship, class variable *y* and input vector values, as $x_{1}$ through $x_{n}$:

(4) $$P\left(y|x_{1},\ldots ,x_{n}\right)=\frac{P\left(y\right)P\left(x_{1},\ldots ,x_{n}|y\right)}{P(x_{1},\ldots ,x_{n})}$$

where *y* denotes class and *x* denotes input values, P(y|x) posterior probability of *y* given the data *x*, P(x|y) probability of input *x* given that the hypothesis was true, P(y) prior probability of *y*, and P(x) is the prior probability of *x*.

Compared to advanced methods, Naive Bayes classifiers can be extremely fast. Each distribution can be estimated independently as a single dimension distribution by decoupling the class-conditional characteristic distribution. On the other hand, this solves problems caused by the dimension curse. NB classier performs efficiently for DDoS detection purpose [41].

#### 3.2.3. Logistic Regression (LR)

LR uses the logistic function to model the binary dependent variable for statistical computations. Many complex extensions of LR have been developed, but the scope of this paper is limited to regression analysis of estimating the binary regression logistic model [42]. In the context of our designed research, the binary logistic model provided an output for the dependent variable to classify the network traffic as normal or DDoS attack and labeled the traffic as 0 and 1, respectively. At the non-linearity nature of data spread, logistic curve fitting is used to posterior probability to execute the binary logistic model. As such, logistic regression model outputs can be interpreted as probabilities of the occurrence of a class. If we use class labels $C^{+}$ and $C^{-}$, the probabilistic output of trained logistic regression model for input *x* will be:

(5) $$P_{C}\left|x^{C^{-}}\right|=\frac{1}{1+exp((x,a)+b)}$$

(6) $$P_{C}\left|x^{C^{+}}\right|=\left(1-P_{C}\left|x^{C^{-}}\right|\right)$$

where the threshold value is for positive labeling (1) is $P_{C}\left|x^{C^{+}}\right|\geq 0.5$ and any value less than this threshold is denoted as zero (0). The implementation of LR classifier for anomaly detection is achieved by [43].

#### 3.2.4. k-Nearest Neighbors (kNN)

kNN is a supervised classification and regression algorithm. The kNN algorithm’s input format is the k nearest training samples in the feature space. Whether kNN is used for classification or regression determines the outcome. This research work concentrates on traffic classification and detection of DDoS attacks. An object is categorised based on a majority vote of its neighbors, with the object being allocated to the class with the most members among its k-nearest neighbors. kNN is a type of instance-based learning, also known as lazy learning, in which the function is only approximated locally and all computation is postponed until after the function has been evaluated. Normalizing the training data can dramatically improve the accuracy of this algorithm, which relies on distance for classification. For DDoS detection, an SDN-enabled network recently used the kNN classifier [44].

#### 3.2.5. Random Forest (RF)

Random decision forests, or RF classification, is an ensemble learning method for classification, regression, and other applications. At training time, RF constructs a large number of decision trees and outputs the class that is the mode of the classes (classification) or the mean prediction (regression) of the individual trees. Decision trees have a tendency to overfit their training set, which is corrected by random decision forests. In [45], anomaly detection has been made through RF classification algorithm.

#### 3.2.6. Ensemble Voting (EV)

EV is a voting classification model which combines the multiple classifiers models into a single model, which is (ideally) stronger than any of the individual models alone. While building a model of ensemble voting, classifier voting was set to “Hard”. Each classifier votes for a class in hard voting, and the class with the most votes wins. Each classifier in soft voting assigns a probability value to each data point that it belongs to a specified target class. In [46], ensemble learning is built over ensemble voting method to achieve more accurate anomaly detection.

### 3.3. Threat Model

In Figure 2 and Figure 3, the host devices are connected to OF switches and act as source and destination for the network actual traffic. Due to the open-access nature of enterprise networks, those hosts can be potentially threatening as they can launch DDoS attacks. To this end, we use Ping3 and Flood command from the command prompt of host devices and targets a particular IP Address of host machine to launch a DDoS attacks. We assume these devices are IoT devices, and network topologies are constructed and managed by Mininet-Wifi. The DDoS attacker can be a remote device on the internet and have access to the local network topology through the middlebox. Our major purpose is to detect and launch mitigation measurements to resolve the network resources for normal network traffic.

## 4. Proposed AMLSDM Framework

The proposed AMLSDN framework’s design is presented in this section. When the network is turned on, the hello messages are exchanged in particular. Those hello messages contain feature-request and feature-reply of OpenFlow protocol exchanged between the SDN controller and OF switches. This exchange procures the overall view of the network in the periodic interval, which is about 30 seconds. Besides, the exchanged hello messages also contain the packets of link layer discovery protocol (LLDP) that depict the overall network topology at the forwarding plane. In the proposed framework, the SDN controller and OF switches are leveraged with the OpenFlow protocol; therefore, to command the network communication, the SDN controller transmits the $OPF_{P}ACKET_{O}UT$, including LLDP, to each OF switch. The proposed framework uses remote POX and Floodlight SDN controllers to coordinate between the control plane and forwarding plane.

**Figure 4:**
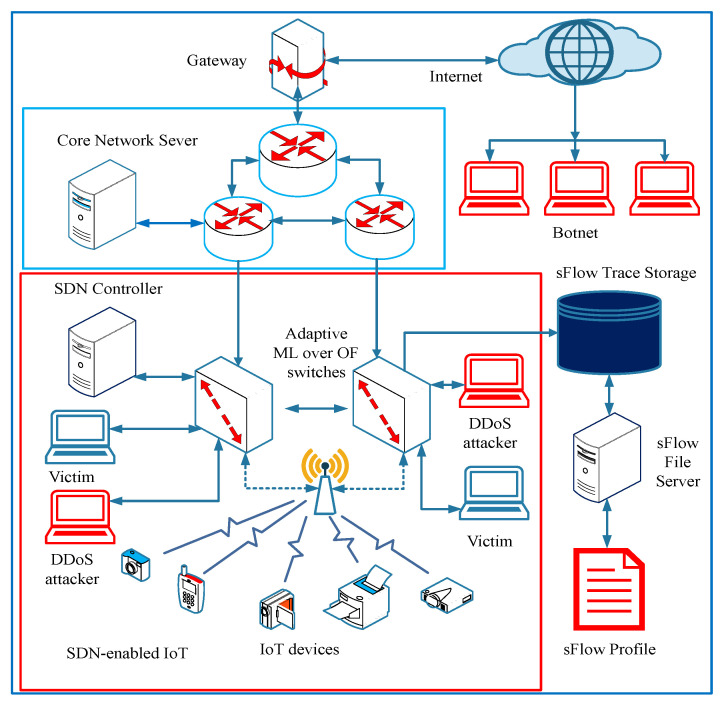
General network architecture to implement adaptive machine learning based DDoS detection.

During the actual communication phase, the different hosts generate network data packets. When data packets from a network flow arrive at the OF switches, the OF switches search their forwarding tables for a matching entry. If a match is detected, the switch forwards the data packet according to the flow table entry’s appropriate action. Otherwise, the switch instructs the controller to calculate the flow’s action. The OF switches are connected with active communication flow and deliver the network topology of all connected OF switches based on the pair of source and destination hosts The active group OF switches shares the flow and port status, including hosts information to controller OFP_PACKET_IN and OFP_PACKET_OUT exchange. The actual network communication can also be generated by the external network source connected through the internet. In this case, OF switches are connected to the core network routers and update the SDN controller about newly arrived network packets. DDoS attacks can be generated within the SDN-enable network domain or by the external network in the form of an individual or grouped (botnet) DDoS attack. Figure 4 describes the general network architecture of the implementation of adaptive machine learning framework for DDoS detection.

To design the DDoS detection and mitigation system for designed network architecture, we divide the functionality of the proposed AMLSDM framework into the following four phases; (i) training the adaptive classification model, (ii) feature extraction of SDN-enabled IoT network traffic phase, (iii) classification of real-time network traffic for DDoS detection phase, and (iv) DDoS mitigation phase.

### 4.1. Training of Adaptive Machine Learning Classification Model Phase

For the development of the adaptive classification model, we utilize the Ensemble Voting (EV) polling system to combine the classification measurement SVM, NB, kNN, LR, and FR machine learning classifiers in a multilayered feed-forwarding manner. At the first layer of the adaptive classification model, the designed module imports SVM, NB, kNN, LR, and FR from Scikit-learn machine learning algorithms python library. The output of the first layer becomes the input for the second layer of the feed-forwarding classification approach. At the second layer, we import the polling classification algorithm of EV, which accumulates the results of trained SVM, NB, kNN, LR, and FR classifiers. After finalizing the adaptive classification model, the adaptive classification module imports the data from an environment-specific dataset. The Pandas library preprocess the data into labeled tabular form and, by utilization of the sklearn.model-selection, split the dataset into the training set and testing set. Then, proposed adaptive classification module initializes the fitting of SVM, NB, kNN, LR, FR, and EV to the training dataset. The EV Classifier is a meta-classifier that uses majority or plurality voting to classify comparable or conceptually different machine learning classifiers. In this paper, we utilize the majority voting of different machine learning classifiers to implement EV Classification. Majority voting based EV classification is further categorized to hard and soft voting classifications. Hard voting entails adding up all of the forecasts for each class label and guessing which one will receive the most votes. Soft voting entails adding up the anticipated probabilities (or probability-like scores) for each class label and predicting the one with the highest probability. In majority voting, EV predicts the class label $\hat{y_{l}}$ of voting of each classifier $C_{j}$ by following equation:

(7) $$\hat{y_{l}}=modeC_{1}\left(x\right),C_{2}\left(x\right),C_{3}\left(x\right),\ldots ,C_{j}\left(x\right)$$

In our case, if the classifiers of SVM, NB, kNN, LR, and FR computes the 0, 1, 0, 1, and 1 values, respectively, then EV predicts the class label as:

(8) $$\hat{y_{l}}=mode\left[0,1,0,1,1\right]=1$$

Simple majority computed by mode most often provides an accurate result to compute class labels. In order to optimize the majority classification of EV, we can optimize the weight $w_{j}$ of a particular classifier by assigning weight values. The weighted majority based voting classification of EV can be computed by following equation:

(9) $$\hat{y_{l}}=margmax_{i}\sum_{j=1}^{J}w_{j}\chi_{L}\left(C_{j}\left(x\right)=i\epsilon L\right)$$

where $\chi_{L}$ is characteristic function and *L* is set of labels. In a weighted ensemble, which is an extension of a model averaging ensemble, the contribution of each member to the final forecast is weighted by the model’s performance. The model weights are small positive numbers, and the sum of all weights equals one, reflecting the percentage of trust or projected performance from each model. Because the weights are uniform, the weighted ensemble functions as a basic averaging ensemble. The weights can be calculated using either each classifier’s rate of accuracy or a holdout validation dataset; there is no analytical approach. As a result, classifiers with a higher accuracy ratio will be favored.

If we assign the $w_{j}$ values to over-selected classifiers such as SVM = 0.1, NB = 0.2, kNN = 0.1, LR = 0.3, and FR = 0.3, then EV predicts the class label of weighted majority as:

(10) $$\hat{y_{l}}=margmax_{i}\left[0.1\times 0,\;0.2\times 1,\;0.1\times 0,\;0.3\times 1,\;0.3\times 1\right]=1$$

The weighted majority classification of adaptive EV provides optimized class labeling for adjustment of prediction according to the characteristic of network traffic. We assign the $w_{j}$ values to all classifiers as SVM = 0.1, NB = 0.2, kNN = 0.1, LR = 0.3, and FR = 0.3 according to the accuracy level of individual classifier. After the completion of the training task of SVM, NB, kNN, LR, FR, and EV, the trained adaptive classification module predicts the final results of DDoS detection by utilizing the testing set. Furthermore, the confusion matrix is prepared to save and analyze the outcomes of the training adaptive classification model. Finally, it imports joblib from sklearn.externals to save the trained adaptive classification model. The output of the second layer becomes the input for the third layer to classify real-time network traffic.

### 4.2. Features Extraction of Network Traffic of Mininet Topology Phase

In AMLSDM framework, the real-time network traffic generated by Mininet network topology experience performance variations after altering the network parameters. We show two different SDN network topologies in Figure 2 and Figure 3 with different network densities and remote SDN controller. However, major traffic features are determined by the rate of packets transmitted over active communication links. Such traffic features determine the difference between normal traffic patterns from a drastic variation of requested network traffic. We develop the traffic extraction module to capture the network traffic and categorized the feature of live network traffic.

Our feature extraction module executes a shell file to collect the traffic statistics over the OF switches. It produces the “SVC” data files of Number of Packets, Size of Bytes, Number of source IPs, and destination IPs. These files are then utilized as run-time input for the designed Python script module, which computes the Rate of Source IP (RSIP), Standard Deviation of Flow Packets (SDFP), Standard Deviation of Flow Bytes (SDFB) [47], Rate of Flow Entries on Switch (RFES), and the Ratio of Pair-Flow Entries on Switch (RP-FES). Network traffic can be manipulated by manual initialization of the DDoS attacks on network hosts to target other particular hosts in the network. This feature extraction module imports NumPy and SVC libraries to compute the following traffic features.

1. Rate of Source IP (RSIP): For a given destination IP address, this function displays the number of source IPs per unit of tim:
  (11) $$\mathrm{RSIP}=\frac{\sum \mathrm{SIP}}{T}$$
  where *T* is the sample time, which can be changed depending on the SDN controller’s ability to handle the traffic flow.
2. Standard Deviation of Flow Packets (SDFP): This is the *T* periodw’s standard deviation for the number of packets:
  (12) $$\mathrm{SDFP}=\sqrt{(\left(1/n\right)*\sum_{i=1}^{n}{\left(packets_{i}-meanPackets\right)}^{2}}$$
  where *n* is the number of active network flows, $packets_{i}$ is the number of packets of flow ith in *T* period, and $packets_{i}$ is the number of packets of flow ith in *T* period, and meanPackets is the average of all flows’ total packets across *T* periods. This feature has a strong link to the occurrence of a DDoS attack because, during an attack, the attacker transmits a large number of attack packets with a small size; these packets will have a significantly smaller standard deviation than typical data packets, resulting in a considerable drop in this parameter.
3. Standard Deviation of Flow Bytes (SDFB): This is the number of bytes in the *T* period’s standard deviation:
  (13) $$\mathrm{SDFB}=\sqrt{(\left(1/n\right)*\sum_{i=1}^{n}{\left(bytes_{i}-meanBytes\right)}^{2}}$$
  where $bytes_{I}$ is the number of total bytes of flow ith in *T* period, while meanBytes is mean of total bytes of all flows in *T* period. SDFB, like SDFP, has a strong link with the occurrence of a DDoS attack, and the expected value of this parameter is lower during an attack than during normal traffic flows.
4. Rate of Flow Entries on Switch (RFES): This is the number of flow entries to the switch per unit of time:
  (14) $$\mathrm{RFES}=\frac{\sum F}{T}$$
  Because the number of flows increases dramatically in a set interval of time during an attack compared to the SFE value during regular traffic flows, this is an important parameter for attack detection.
5. The Ratio of Pair-Flow Entries on Switch (RPFES): The total number of flows in the *T* period divided by the number of interactively divided flow entries in the switch:
  (15) $$\mathrm{RFES}=\frac{\mathrm{IntIP}}{N}$$
  where *N* is the total number of IPs and IntIP is the total number of interactive IPs in the flow. As a result, as soon as the attack begins, the number of interaction flows will drop dramatically.

After the computation of the above features, the feature extraction module creates the header of RSIP, SDFP, SDFB, RFES, and RPFES and assigns the computed values. On behalf of the calculated values, the feature extraction module labeled the normal traffic as 1. Finally, the feature extraction module creates the training data file of “live.csv” for the newly arrived network traffic to accommodate the real-time network traffic. This file “live.csv” only keeps the entry of newly computed network traffic and serves as the input to the trained adaptive machine learning model. These five network traffic features, Rate of Source IP (RSIP), Standard Deviation of Flow Packets (SDFP), Standard Deviation of Flow Bytes (SDFB), Rate of Flow Entries on Switch (RFES), and the Ratio of Pair-Flow Entries on Switch (RPFES), are efficient enough to differentiate between DDoS attacks and normal traffic. According to these features, DDoS attacks show abnormality in an exponential increase in the values of Rate of Source IP (RSIP), Standard Deviation of Flow Packets (SDFP), Standard Deviation of Flow Bytes (SDFB), Rate of Flow Entries on Switch (RFES), and the Ratio of Pair-Flow Entries on Switch (RPFES). In this way, DDoS detection becomes much more accurate and efficient.

### 4.3. Classification of Real-Time Network Traffic Phase

In this phase, we implement our designed inspection module to classify the network traffic being arrived at real-time data entry of live.csv. This inspection module imports all the python libraries needed for trained adaptive machine learning models and live.csv. After importing all required libraries, it loads the trained adaptive machine learning model and real-time network database of live.csv. Finally, this module predicts the results from incoming data eateries. The computed features are combined to determine if an interaction flow is normal traffic or under DDoS attack. In DDoS attacks, the number of flow entries to a certain destination host increases dramatically over time *T*, and the destination host is unable to respond to network traffic requests. As a result, as soon as the attack begins, the number of interaction flows will drop dramatically. To make this detection parameter scalable to the network under various operating conditions, the total number of interactive flows is divided by the total number of flows. By using these parameters, the AMLSDM framework utilize the trained adaptive machine learning model and continues the inspection of incoming flows to classify the DDoS attacks from normal network traffic. This DDoS detection is being made over the third layer of the proposed multi-layered feed-forwarding framework.

### 4.4. Mitigation of DDoS Attacks Phase

Our proposed AMLSDM framework executes the inspection modules of classifications overall active OF switches and detects the DDoS attacks actively for run-time traffic. The OF switches update the SDN controller periodically about flow-tables and traffic rate over ingress and egress active posts to report the detection DDoS attacks. The remote SDN controller executes the built-in congestion control mechanism module to mitigate the DDoS attacks. The remote SDN controller commands the active OF switches to drop the specific communication request and switch the network resources to the legitimate network hosts. OF switches follow the instructions of the SDN controller and discard the traffic request of specific source hosts after investigated as DDoS attacks. In this way, the network resources are switched to legitimate network users to carry on the normal network traffic.

**Figure 5:**
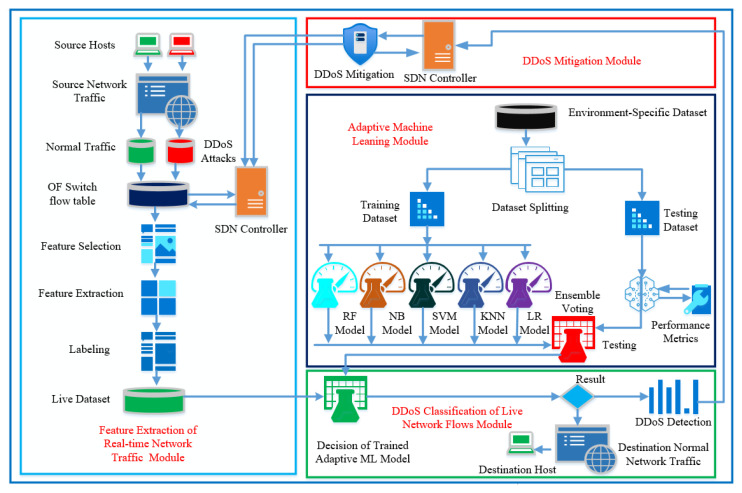
Flowchart and work mechanism of DDoS detection and mitigation system of AMLSDM.

The operation of the above four phases results in the execution of designed four modules to achieve the DDoS detection and mitigation of the proposed AMLSDM framework. The detailed flowchart and work mechanism of the proposed AMLSDM framework is shown in Figure 5.

## 5. Experiment Setup

Different forms of normal traffic were mixed in with the attack traffic, and the parameters of the attack flow were varied. Oracle VirtualBox, Mininet, Mininet-wifi, Open vSwitch, POX Controller, Floodlight Controller, Miniedit, tshark, and sFlow-RT are among the applications that must be installed. We run the Pox and Floodlight controller at Virtualbox and run the sFlow-RT on our host Linux machine to capture the network traffic generated by Mininet SDN network topologies. We design a Python script to develop the adaptive classification module to integrate all the required libraries and packages to build a trained adaptive classification model. This module uses core Numpy and Pandas Python libraries for data processing. For the execution of real-time Mininet network topology for network simulation, this module also imports the default-timer and DateTime libraries. For data visualization of simulation performances, it also imports matplotlib.pyplot library. The adaptive classification module also needs performance metrics of Classification Report, Accuracy Score, Confusion Matrix, Recall Score, Precision Score, and F1 Score Python libraries to show the outcomes of the classification.

We launch the threat model of DDoS described earlier and monitor the performance variation by sFlow-RT. Then we launch our machine learning-based inspection model as collect.sh, which classifies the network traffic as normal or DDoS attacks and mitigates the effect by intelligent resource distribution towards legitimate hosts. We validate our proposed framework with extensive simulations experiments, but for this paper, we report the simulation results of SDN-enabled IoT network topologies shown in Figure 2 and Figure 3.

### 5.1. Dataset Description

The proposed AMLSDM framework leverages an environment-specific dataset on which the evaluations are performed. The network statistics are converted to a “CSV” file in this environment-specific dataset. The terminal-based program named “tshark” is used to generate that CSV file with limited fields. Moreover, the above-mentioned dataset is further categorized into traffic and attack datasets which are shown in the following Table 1 and Table 2, where the maximum variations can be seen in the packet interval field between the normal and attack traffic.

**Figure 6:**
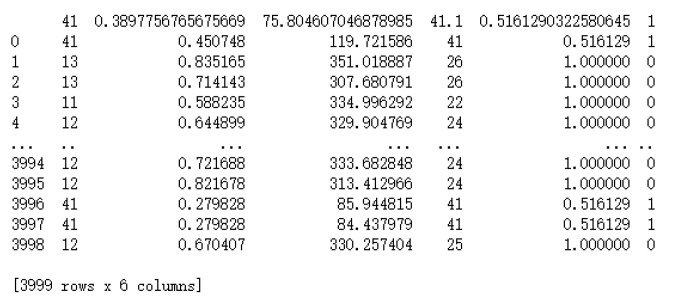
Print of the overall environment-specific dataset over normal or DDoS attach.

Figure 6 shows the print of the overall dataset demission, which contains 3999 rows and 6 columns. Based on initial parameters, the last column computes the traffic classification as a normal or DDoS attack.

### 5.2. Experiments Results

We simulate the AMLSDM framework for two different MININET network topologies with POX and floodlight SDN controllers, respectively. The proposed AMLSDM framework performs the DDoS detection and mitigation with an adaptive machine learning classification model with support of the voting polling system of EV, which combines SVM, NB, kNN, LR, and RF.

#### 5.2.1. Experiments Results for DDoS Detection and Mitigation of AMLSDM

**Figure 7:**
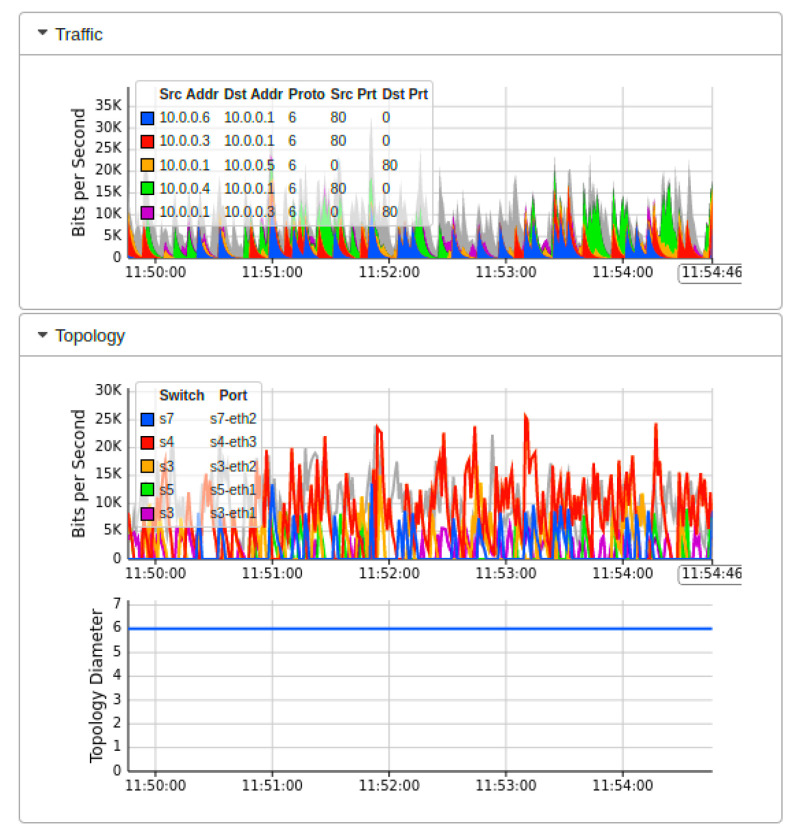
Mininet performance dashboard during real-time normal traffic (T1).

**Figure 8:**
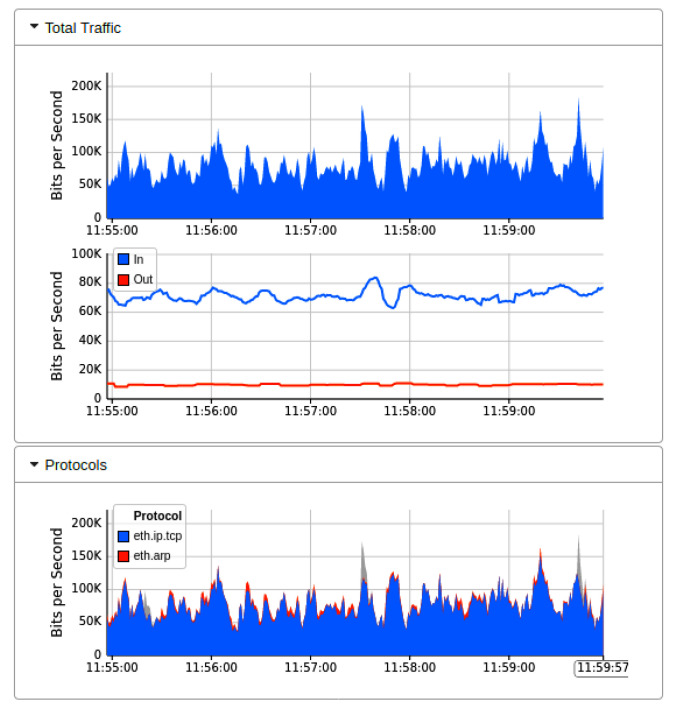
Network bandwidth dashboard during real-time normal traffic (T1).

**Figure 9:**
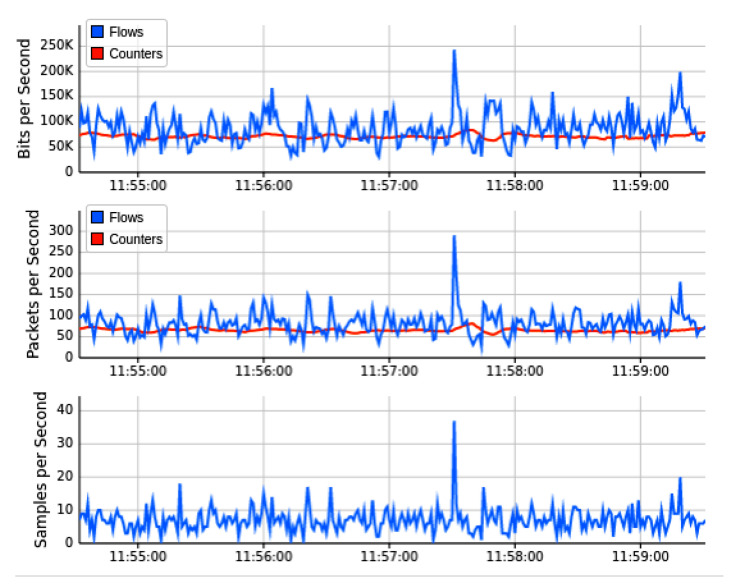
Performance dashboard for OF switches during normal traffic (T1).

Figure 7 showed the real-time MININET emulation performance dashboard captured by sFlow for the proposed AMLSDM framework of the proposed network topology as shown in Figure 2. Those simulation settings are using POX SDN controller in a control plane. The SDN-enabled IoT network topology comprises of 14 OF Switches, that supports 28 end hosts. This network topology uses the external SDN controller of POX, which is running on VirtualBox. The ICMP ping messages are exchanged between the nodes to verify the reachability of the connected hosts in the network. The purpose of those ping messages is to check whether all the nodes are successfully linked with the network topology or not. Moreover, those ping messages also verify the regular network traffic by the nodes. The result of the successful link indicates that there is no malicious node present in the network, and a DDoS attack has not been initiated up to this point in the simulations. To start ICMP ping, we execute a traffic test over specific hosts, which checks the reachability of each network host individually. The sub-graph of traffic indicates the IP address of sources hosts and currently replying to destination hosts. In the absence of a DDoS attack, the normal traffic reaches a maximum of 30Kbps. The sub-graph of bits per second shows the particular OF switches and their associated ports involved in the current network traffic transmission. The subgraph of topology diameters indicates the traffic generation of particular host devices. Figure 8 indicates the network bandwidth dashboard in terms of bits per second utilized by the current generated network traffic. TCP and ARP protocols are being utilized for the basic ICMP network reachability test. Meanwhile, Figure 9 indicates the sFlow-test dashboard to identify the performance of switches’ activities regarding current simulation testing. The involved switches analyze the simulation by counting the flows, check sequence number, comparing byte flows to counters, comparing packet flows to counters, and checking the ingress and egress ports information and CPU load average to identify the nature of current traffic. We run the Collect.sh script to collect this information for machine learning algorithms based inspection to identify the DDoS attacks. Figure 9 provided the evidence that network traffic is normal, and our designed inspection validates the normality of the current network traffic.

**Figure 10:**
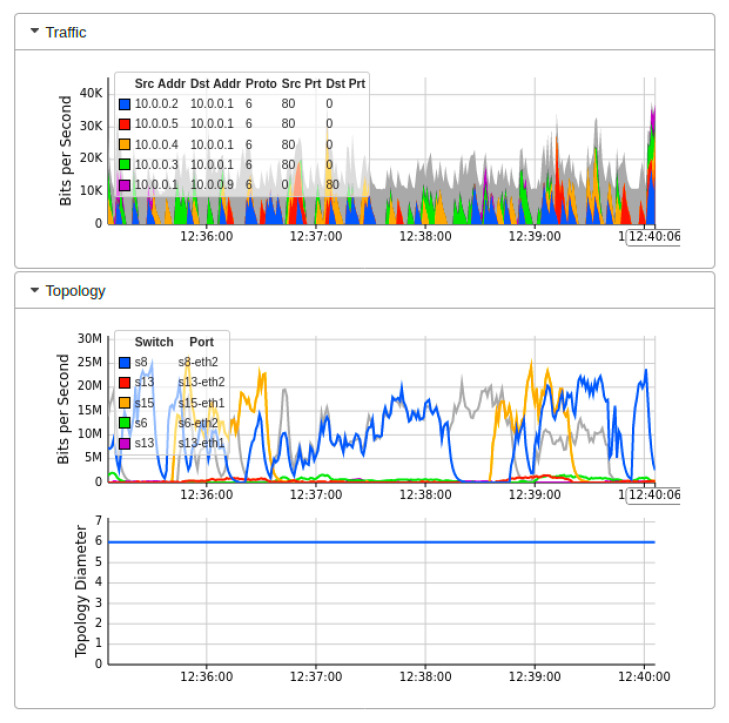
Mininet performance dashboard during real-time DDoS attacks (T1).

**Figure 11:**
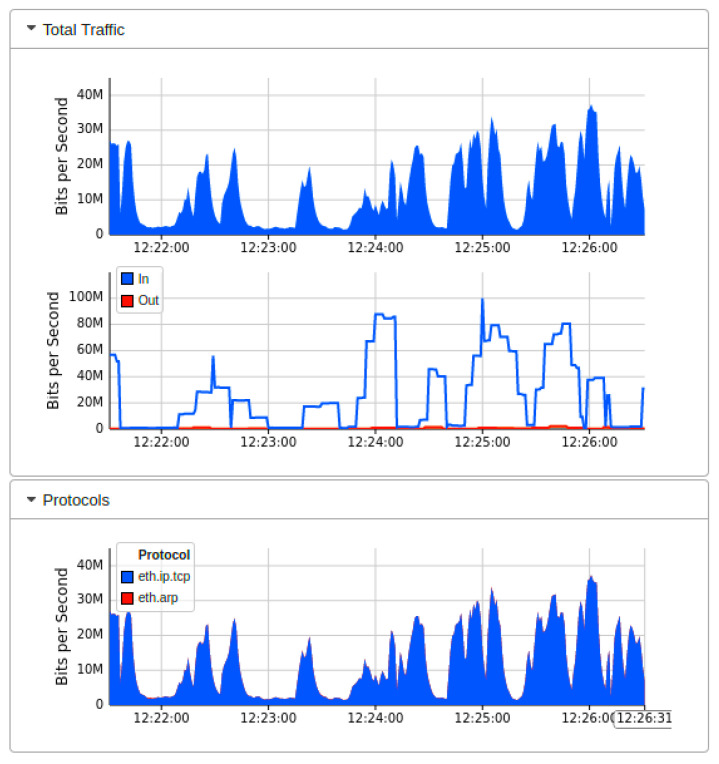
Network bandwidth dashboard during real-time DDoS attacks (T1).

**Figure 12:**
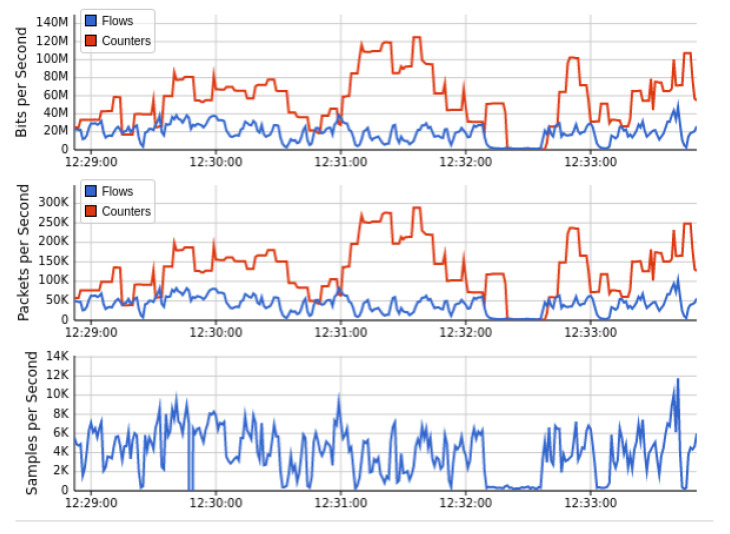
Performance dashboard for OF switches during DDoS attacks (T1).

Figure 10 showed the output results when we launch the DDoS attack in our current Mininet network topology under the implementation of Floodlight SDN controller. We launch the DDoS attack by utilizing the thread model earlier discussed. The successful network traffic of sub-graph of Figure 10 indicates that the performance of the network is affected by the DDoS attacks initially. Still, our AMLSDM mitigation system stabilized the network performance even in the presence of DDoS attacks. Our proposed AMLSDM rescheduled the network resources to legitimate hosts and discarded the DDoS traffic requests. The sub-graph of the network topology shows the rate of the requested network traffic increased exponentially and 30 Kbps traffic rise to 30 Mbps. This increment of the requested traffic rate is due to DDoS attacks, and it keeps rising with the passage of the time. Similarly, Figure 11 showed the bandwidth utilization of the overall network traffic, which is drastically higher than normal network traffic. This sub-graph also shows the DDoS detection and mitigation effect of the proposed AMLSDM framework, which successfully classifies the normal traffic from DDoS and switches the network resources. Therefore, in simulation segments, we can see the higher spikes of the captured network bandwidth up to 40 Mbps, and then AMLSDM reduces it to back to normal 30 Kbps to 50 Kbps. Similarly, Figure 12 of the sFlow-test dashboard captured the performance fluctuation over network switches and identifies the increment of traffic load. We can observe the exponential growth of requested network traffic as the simulation duration increases. In the response, we can see the successful effect of AMLSDM implementation, and network traffic goes down in segments when the proposed framework mitigates the impact of DDoS attacks. The high spikes of data flows show the network traffic caused by the DDoS attacks, meanwhile low spikes indicate the effect of AMLSDM implementation, which successfully reduced the traffic over OpenFlow switches by detecting and mitigating the attacks. This process is continued during the whole simulation period. During the simulation period, DDoS attacks damage the network performance by capturing the maximum resources, meanwhile the designed AMLSDM identifies the attack and restores the network performance.

**Figure 13:**
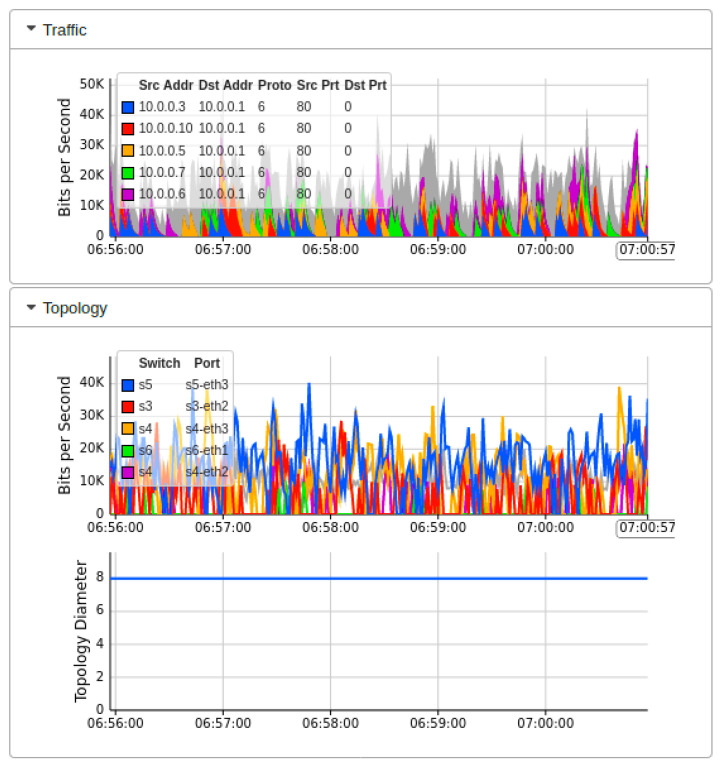
Mininet performance dashboard during real-time normal traffic (T2).

**Figure 14:**
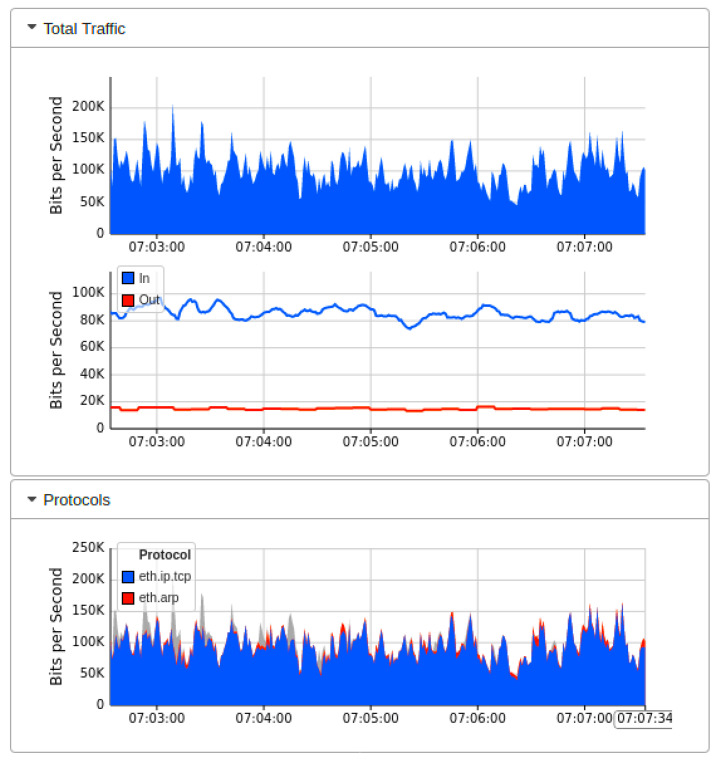
Network bandwidth dashboard during real-time normal traffic (T2).

**Figure 15:**
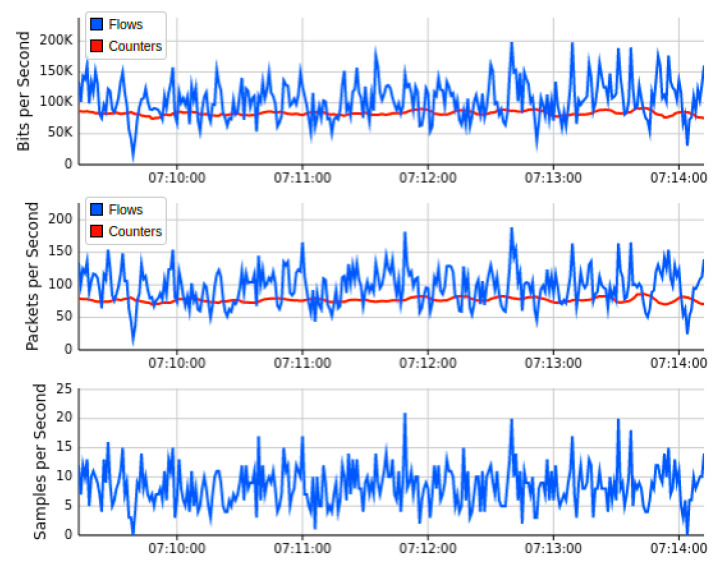
Performance dashboard for OF switches during normal traffic (T2).

Figure 13 shows the outcome of the second network topology, in which the SDN controller of floodlight is connected to 32 OF switches in tree topology manner. Each of these 32 switches provides connectivity to two IoT hosts on the forwarding plane. The real-time Mininet emulation performance Dashboard presents the sub-graph of the network traffic, which shows the source IP addresses on which we run the test-script of ping-all. Normal network traffic of each host reaches 40 Kbps maximum, and overall network resources captured are minimal at this stage of simulation. Similarly, the subgraph of network topology indicates a similar traffic trend over currently involved OF switches. The topology diameter sub-graph shows the number of host devices from where we launch the ping-all task. The periodic outcome of this graph indicates the normal pattern of network traffic, in which all devices are able to ping smoothly and all OpenFlow switches are available to forward the network traffic of forwarding plane. Figure 14 indicated the bandwidth utilization parameters over OF switches captured by the sFlow bandwidth dashboard. Sub-graphs of Figure 14 indicated the normal trend for network traffic on egress and ingress ports of switches. Similarly, Figure 15 monitored the simulation analysis over the specific experiment duration. Sub-graphs of these results show bits per second, packets per second, and sample per second of network traffic OF switches, and identify the presence of malicious traffic. Our AMLSDM framework use collect.sh script over all the switches to inspect the traffic according to trained machine learning models. The inspection results validate the normality of traffic at this stage of simulation.

**Figure 16:**
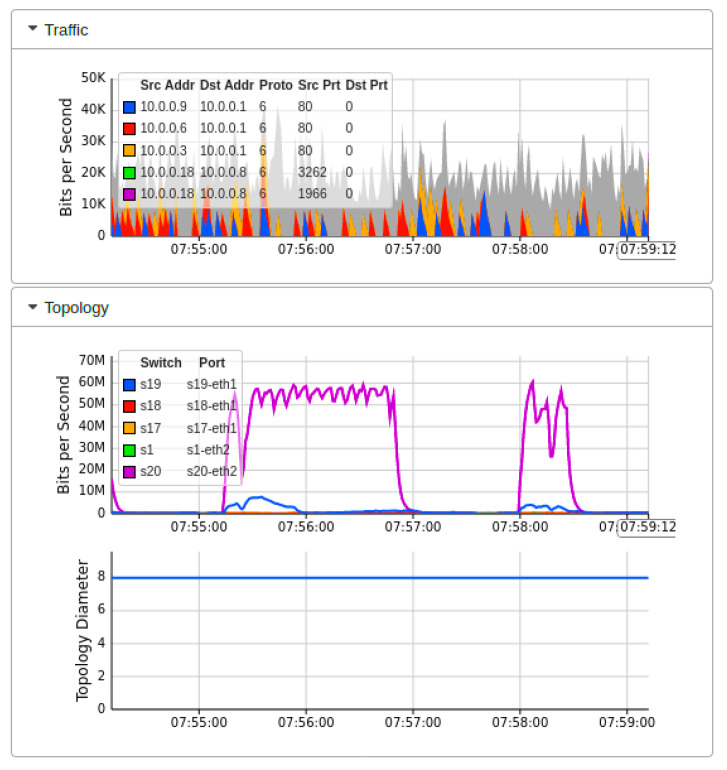
Mininet performance dashboard during real-time DDoS attacks (T2).

Figure 16 showed the performance fluctuation once we launch the DDoS attack through the threat model. The threat model starts flooding ICMP messages to specific devices with a faster request rate. As a result, successful network traffic is poorly affected. The sub-graph of network topology shows the exponential growth of traffic requests over OF switches, which dramatically increase the traffic to 70 Mbps over a single OF switch. Meanwhile, the network diameter remains constant, but participating devices now face the flooding of ICMP messages at a drastic rate. As a result of this DDoS attack, the network throughput performance could reach zero, but due to the implementation of the AMLSDM framework, the network performance is stabilized with the passage of time.

**Figure 17:**
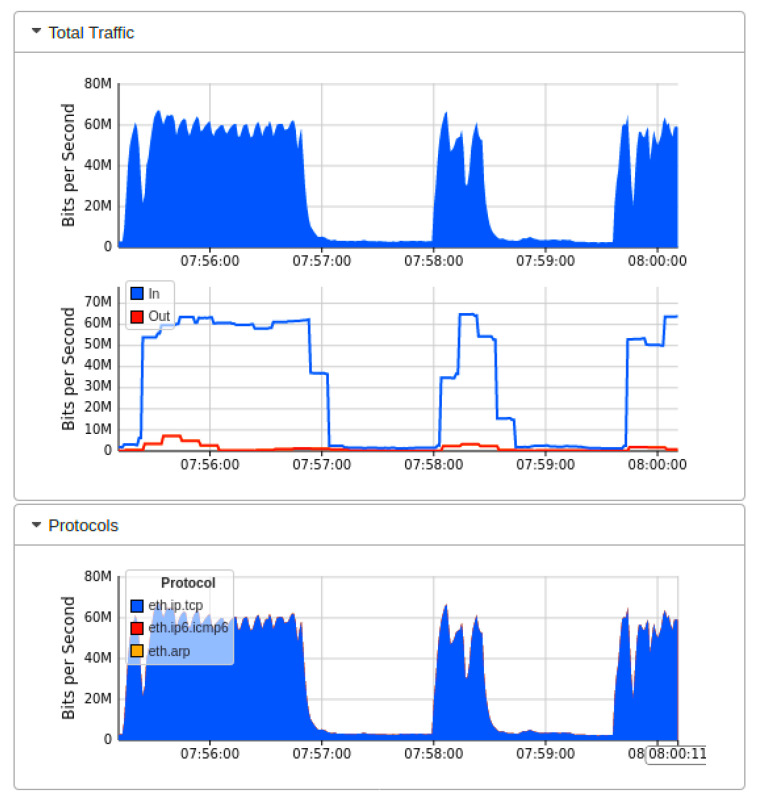
Network bandwidth dashboard during real-time DDoS attacks (T2).

**Figure 18:**
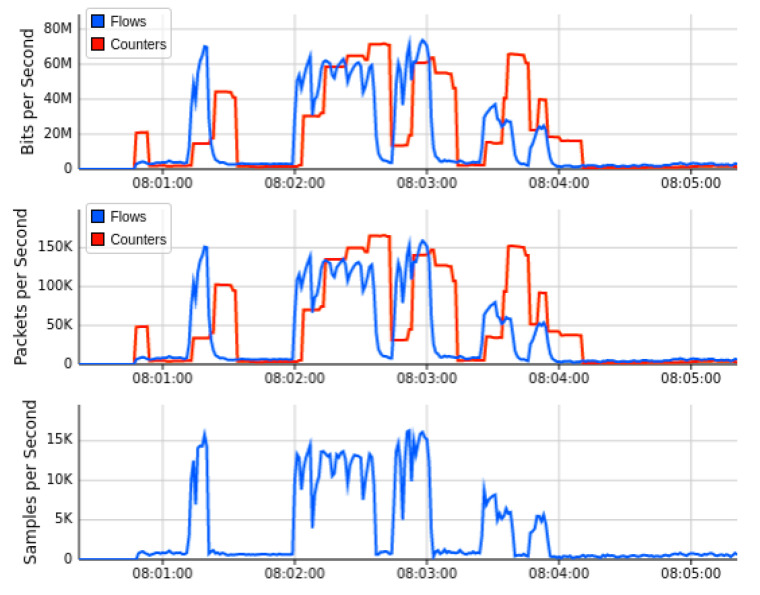
Performance dashboard for OF switches during DDoS attacks (T2).

Similarly, Figure 17 and Figure 18 indicated the performance fluctuation at particular simulations segments when DDoS attacks are in action, and also indicate the performance restoration with the help of DDoS detection and mitigation of AMLSDM framework. Figure 17 showed network bandwidth utilization dashboard during real-time DDoS attacks. We can observe that network traffic increases exponentially once the DDoS attacks are being generated. Once we launch the AMLSDM framework, the network traffic decreases dramatically, and most of the DDoS are being detected and mitigated. In this way, we are able to give the resources back to legitimate users. Meanwhile, Figure 18 indicated the performance dashboard for OF switches during DDoS attacks. We experience the DDoS attacks over OF switches during simulation period, which causes the performance issue for OF switches. We execute the AMLSDM framework to bring down the network traffic to normal behavior.

#### 5.2.2. Features Engineering Results

**Figure 19:**
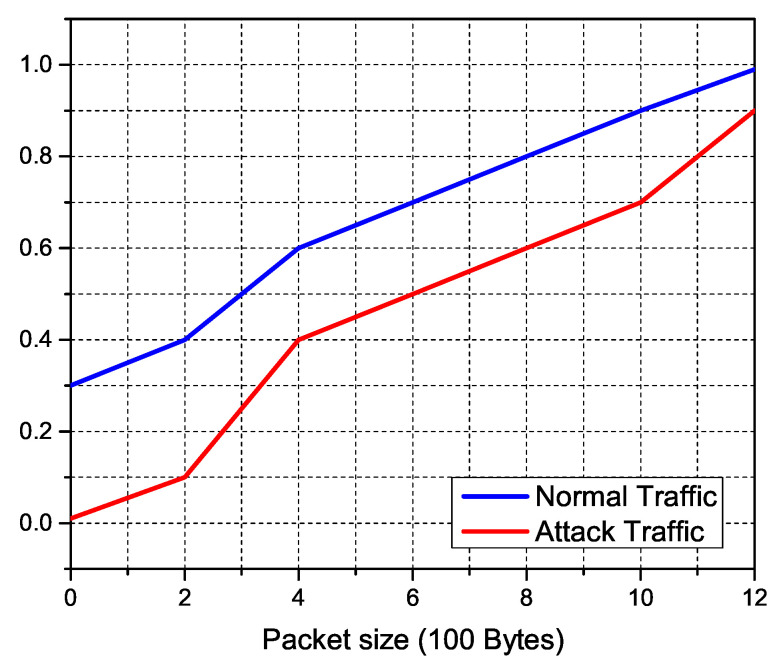
Detecting DDoS anomalies using IoT traffic feature-statistics based on packet sizes.

**Figure 20:**
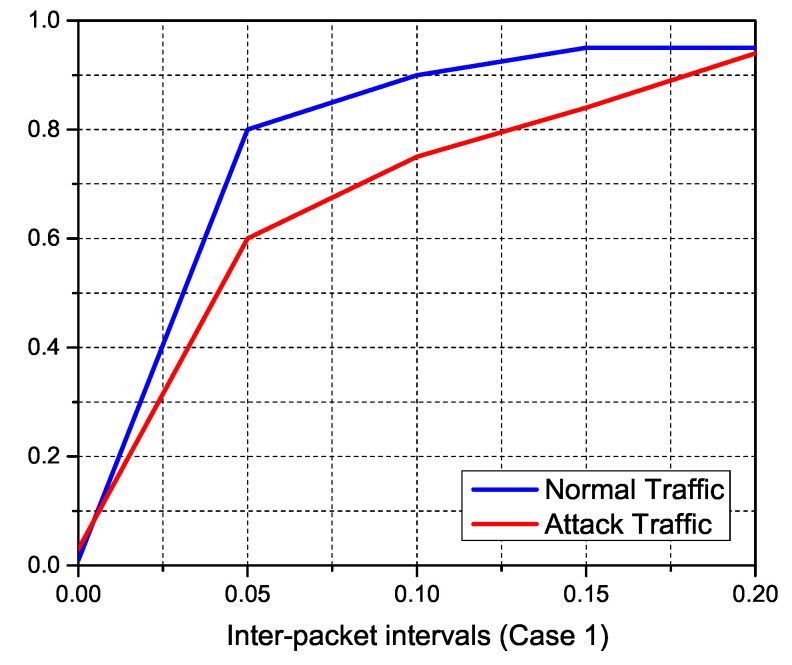
The inter-packet intervals of ΔT are used to detect DDoS anomalies in IoT traffic.

**Figure 21:**
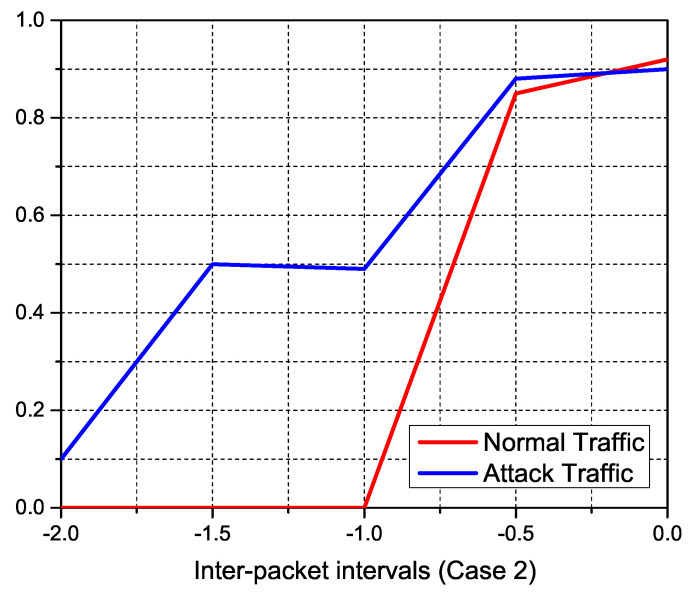
DDoS anomaly detection from IoT traffic feature-statistics according to the inter-packet intervals of $\frac{d\Delta T}{dt}$.

**Figure 22:**
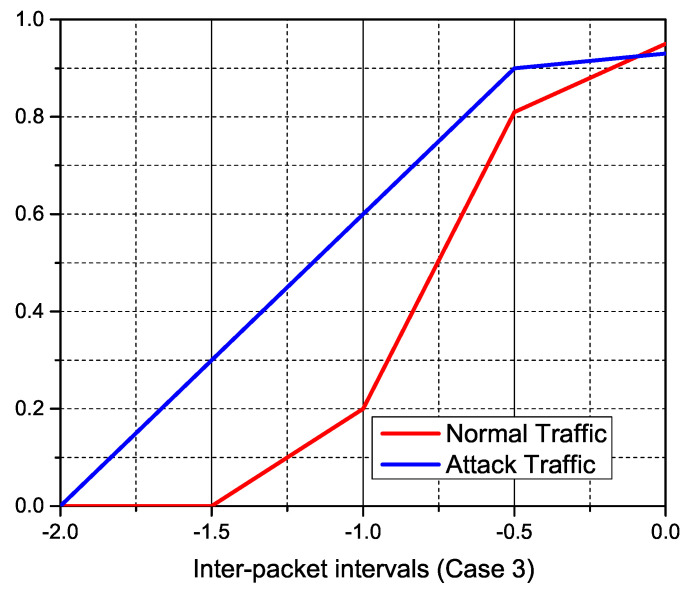
DDoS anomaly detection from IoT traffic feature-statistics according to the inter-packet intervals of $\frac{d^{2}\Delta T}{dt^{2}}$.

We study the features of the resultant traffic using our simulations, which are displayed in the accompanying figures, to inspect typical IoT traffic and anonymous DDoS attack traffic. The incoming traffic stream was successfully separated towards the sFlow database using pre-defined traffic features of expected normal and attack traffic. The size of normal traffic packets versus attack packets is depicted in the Figure 19. On the other hand, regular traffic packets range in size from 100 to 1200 bytes, with over 90% of assault packets being under 100 bytes. To obtain control of accessible connections, DDoS attacker tools often flood the network with tiny TCP and SYN queries. Figure 20, Figure 21 and Figure 22 indicate the traffic inter-packet interval feature to check the synchronous behavior of requested traffic. Authorized IoT-enabled devices send traffic requests at preset intervals, whereas assaults happen at random. DDoS attacks use small packet sizes and a short packet interval to interrupt internet flow. The effect of DDoS attacks on traffic over ΔT and its first and second level derivatives of packet intervals is confirmed by these figures. Similarly, $\frac{d\Delta T}{dt}$ and $\frac{d^{2}\Delta T}{dt^{2}}$ inter-packet intervals feature support classifiers to identify the difference between normal and DDoS attack.

#### 5.2.3. Classification Performance of DDoS Detection for Adaptive Machine Learning Model

We perform extensive simulations of the AMLSDM framework with an EV trained model and also simulate the AMLSDM framework with a stand-alone trained model of SVM, NB, kNN, LR, and RF to compare the performance enhancement of adaptiveness of the proposed framework. We refer to these frameworks as AMLSDM-EV, AMLSDM-SVM, AMLSDM-NB, AMLSDM-kNN, AMLSDM-LR, and AMLSDM-RF. We capture the classification measurements of all frameworks for SDN-IoT network topology of Mininet for lengthy simulation periods to test 5000, 1000, 15,000, 20,000, 25,000, and 30,000 network flows entries in the “live.csv” dataset. DDoS detection dashboard of sFlow returns the periodic performance metrics of Accuracy, Precision, Recall, and F1 Score. These performance metrics are derived from the following factors; True Positive (TP), True Negative (TN), False Positive (FP), and False Negative (FN). The following equation computes the accuracy:

(16) $$\mathrm{Accuracy}=\frac{\mathrm{TP}\;+\;\mathrm{TN}}{\mathrm{TP}\;+\;\mathrm{TN}\;+\;\mathrm{FP}\;+\;\mathrm{FN}}$$

**Figure 23:**
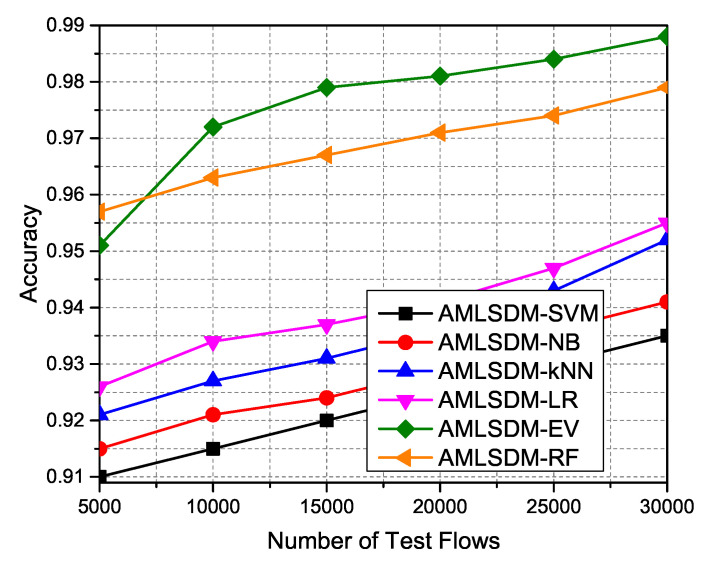
Accuracy performance of ML classifiers.

Figure 23 indicates that the adaptive AMLSDM-EV outperformed the static AMLSDM-SVM, AMLSDM-NB, AMLSDM-kNN, AMLSDM-LR, and AMLSDM-RF models. As the simulation duration increases, the overall performance of the adaptive machine learning-based AMLSDM-EV framework is improved in terms of the classification property of the DDoS framework. The major reason for this periodic improvement is due to the systemic improvement of the accuracy of the adaptive machine learning classification model. Among all other classifiers, the ascending order of achieved accuracy is as follows; AMLSDM-SVM, AMLSDM-NB, AMLSDM-kNN, AMLSDM-LR, and AMLSDM-RF. Meanwhile, the precision is computed by the following equation:

(17) $$\mathrm{Precision}=\frac{\mathrm{TP}}{\mathrm{TP}\;+\;\mathrm{FP}}$$

**Figure 24:**
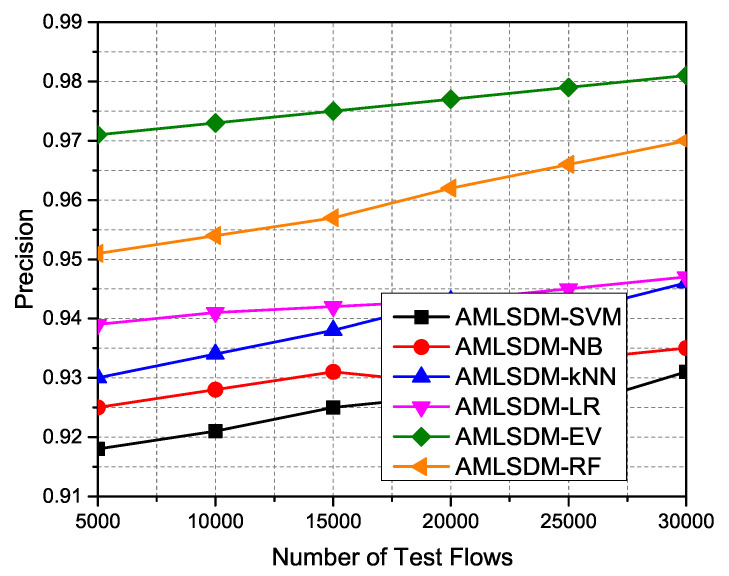
Precision performance of ML classifiers.

With higher accuracy, higher precision is also critical to understand the consistency of the designed framework. Figure 24 elaborates on the precision results of periodic simulation over different time duration. From this result, we can derive the fact that the overall precision of adaptive AMLSDM-EV also improves along with accuracy. However, the utilization of different classification algorithms provides minor performance fluctuations. As we witnessed the earlier better performance of the AMLSDM-EV in the case of higher accuracy, AMLSDM-EV similarly provides maximum precision as compared to AMLSDM-SVM, AMLSDM-NB, AMLSDM-kNN, AMLSDM-LR, and AMLSDM-RF trained models. To compute the correct positive cases from all the positive cases, the performance metric Recall is used, and the following equation can compute it as follows:

(18) $$\mathrm{Recall}=\frac{\mathrm{TP}}{\mathrm{TP}\;+\;\mathrm{FN}}$$

**Figure 25:**
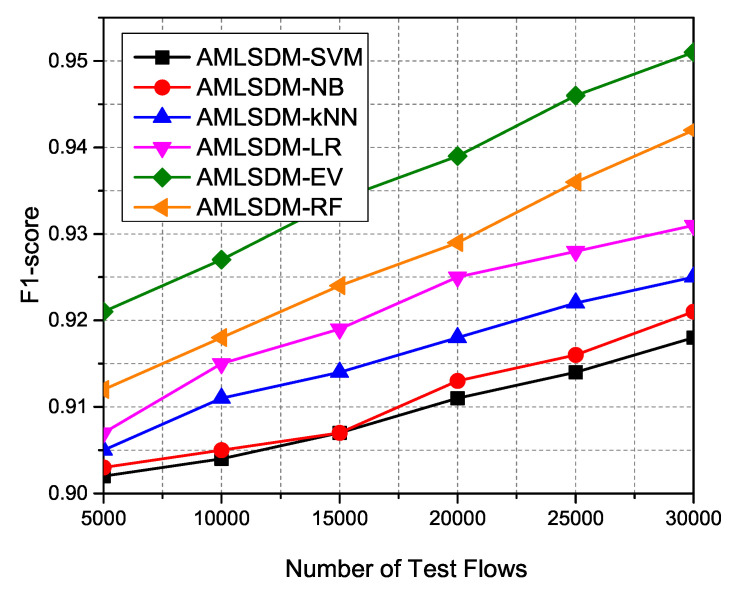
F1-score performance of ML classifiers.

Figure 25 indicates the performance metric of Recall outcomes in the case of all the used machine learning classiers in the proposed AMLSDM framework. Simulation results validate the better performance of AMLSDM-EV over AMLSDM-SVM, AMLSDM-NB, AMLSDM-kNN, AMLSDM-LR, and AMLSDM-RF models. The major reason for this continuous better performance of the AMLSDM-EV model is due to the required critical examination of real-time network execration and real-time network traffic generation for both normal and DDoS attacks. Run-time execution of the adaptive AMLSDM-EV classification model combines the verdicts of SVM, NB, kNN, LR, and RF classifiers to become suitably designed for the detection and mitigation setup of the AMLSDM framework. In order to reduce the False Positive of the classification, the performance metric F1 Score is used and computed by the following equation:

(19) $$F1-\mathrm{score}=2*(\frac{\mathrm{Precision}*\mathrm{Recall}}{\mathrm{Precision}+\mathrm{Recall}})$$

**Figure 26:**
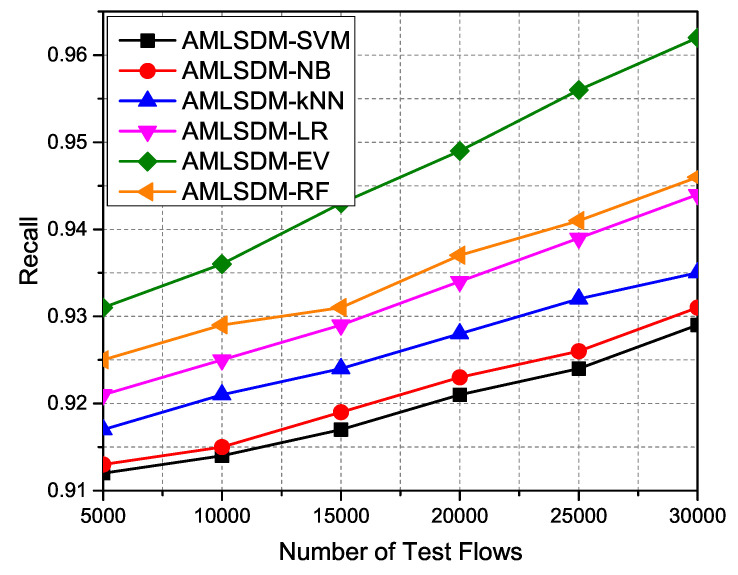
Recall performance of ML classifiers.

F1 Score is the harmonic mean of Precision and Recall and correctly identifies the False Positive rate. Figure 26 indicated the performance outcomes of the F1 Score metric for all the AMLSDM-EV, AMLSDM-SVM, AMLSDM-NB, AMLSDM-kNN, AMLSDM-LR, and AMLSDM-RF classification configurations of the proposed AMLSDM framework. The AMLSDM framework with adaptive machine learning-based AMLSDM-EV performs more efficiently than other all machine learning classifiers used in our simulation setup.

#### 5.2.4. Performance Comparison with Different State-of-the Art Solutions

**Figure 27:**
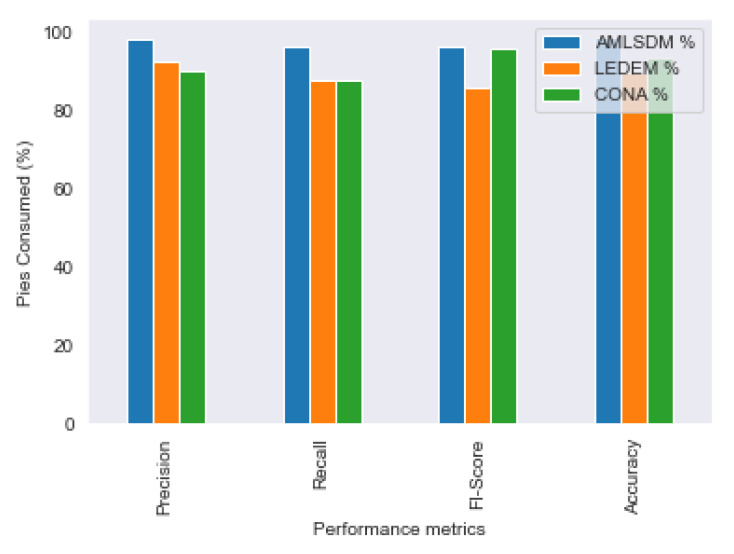
Performance comparison of AMLSDM with LEDEM and CONA.

Figure 27 shows the performance comparison of the proposed AMLSDM with state-of-the-art solutions, such as LEDEM [34] and CONA [19]. Our proposed AMLSDM framework provides better performance for all performance metrics of Accuracy, Precision, F1 Score, and Recall. The major edge of the proposed solution is an adaptive machine learning classification model to detect DDoS attacks.

## 6. Conclusions

In this paper, we proposed an AMLSDM framework based on an adaptive machine learning classification model to detect DDoS attacks for network traffic of SDN-enabled IoT. The AMLSDM framework also provides a DDoS mitigation system to switch network resources to normal network traffic. The multilayered feed-forwarding design of the AMLSDM framework utilizes SVM, NB, kNN, LR, and FR classifiers in the first layer. The output of the first layer is provided as input to the second layer EV, which accumulates the performance of first layer classifiers to detect the DDoS attacks. The trained adaptive machine learning model predicts the DDoS attacks for real-time network traffic at the third layer. We implement our proposed framework in four phases; (i) training the adaptive classification model, (ii) feature extraction of SDN-enabled IoT network traffic phase, (iii) classification of real-time network traffic for DDoS detection phase, and (iv) DDoS mitigation phase. In every phase, we execute the adaptive classification module, feature extraction module, DDoS inspection module, and DDoS mitigation module. Our extensive simulation results validate the intelligent DDoS detection and mitigation of the AMLSDM framework to classify the real-time network traffic generated by two SDN-enabled IoT networks. Performance metrics of Accuracy, Precision, Recall, and F1 Score validate the better classification of adaptive setting AMLSDM-EV as compared to static AMLSDM-SVM, AMLSDM-NB, AMLSDM-kNN, AMLSDM-LR, and AMLSDM-RF classification configurations. We also perform the simulation comparison with state-of-the-art LEDEM and CONA frameworks to validate the better performance of the AMLSDM framework.

In the future, mitigation of DDoS attacks at SDN controller will be explored more deeply, as it is one of the key research area to further improve the utilization of SDN controllers in IoT networks. We will also extend the implementation of our proposed framework to detect phishing attacks. 

## Figures and Tables

**Table 1:** Characteristics of the traffic features.

RSIP	SDFP	SDFB	RFES	RPFES
41	0.450748	119.721586	41	0.516129
41	0.279828	85.944815	41	0.516120
41	0.278636	84.437979	41	0.516129

**Table 2:** Characteristics of the DDoS attacks features.

RSIP	SDFP	SDFB	RFES	RPFES
12	0.721688	333.682848	34	1.00000
12	0.821678	313.412966	24	1.000000
12	0.6704407	320.257404	25	1.000000
